# Characteristics of Locomotive Syndrome in Japanese Patients with Chronic Pain and Results of a Path Analysis Confirming the Relevance of a Vicious Cycle Involving Locomotive Syndrome, Musculoskeletal Pain, and Its Psychological Factors

**DOI:** 10.31662/jmaj.2019-0009

**Published:** 2019-08-06

**Authors:** Michiko Ushio, Masahiko Sumitani, Hiroaki Abe, Kazuhito Mietani, Jun Hozumi, Reo Inoue, Rikuhei Tsuchida, Takahiro Ushida, Yoshitsugu Yamada

**Affiliations:** 1 Department of Anesthesiology and Pain Relief Center, The University of Tokyo Hospital, Tokyo, Japan; 2 Department of Pain and Palliative Medicine, The University of Tokyo Hospital, Tokyo, Japan; 3 Multidisciplinary Pain Center, School of Medicine, Aichi Medical University, Aichi, Japan

**Keywords:** musculoskeletal pain, locomotive syndrome, vicious cycle, psychological distress

## Abstract

**Introduction:**

The current aging population has a major impact on public health. Locomotive syndrome (LS) is a condition that carries a high risk for developing systemic musculoskeletal disability.

**Methods:**

Participants were patients with chronic pain (n = 415) who were examined at the Japanese multidisciplinary pain centers of the research consortium. They completed the 25-question geriatric locomotive function scale (GLFS-25; LS screening tool), an 11-point numerical rating scale (NRS) of pain intensity and its psychological distresses, health-related quality of life (HRQOL) questionnaire, and a survey of exercise habits. A multifaceted analysis of the relevance of the pain, psychological distresses, and LS were conducted using SPSS and AMOS software.

**Results:**

337 patients (81.2%) were found to have LS. The final model of a multifaceted analysis demonstrated good fitness for the “vicious cycle” model among the results of LS, pain intensity, impairment of self-efficacy, and depression; these parameters independently impaired HRQOL. Anxiety related to falling (GLFS-25) and exercise habits affected the model.

**Conclusions:**

These findings indicate LS, LS-related pain, and psychological distress create a vicious cycle, resulting in the impairment of HRQOL. Treatment strategies for LS should inclusively focus on musculoskeletal disorders, pain, and pain-related psychological factors.

## Introduction

The current aging population has a major impact on public health in Japan. In 2015, people aged 65 years or older accounted for 25% of the Japanese population. Currently, there are approximately 5 million people who require nursing care services. Locomotive syndrome (LS) is a condition present in late middle-aged to elderly population who are at high risk of developing systemic musculoskeletal disability ^(1), (2)^. Candidates with LS are estimated to require nursing care services as a result of musculoskeletal disorders. The prevention and the treatment for the LS are definitely important for extending healthy life expectancy in the super-aged society.

The 25-question geriatric locomotive function scale (GLFS-25) was developed to screen for and evaluate the severity of LS ^(3)^. The GLFS-25 is a patient-based questionnaire that quantitatively measures impairment in musculoskeletal function. It includes items related to locomotor ability and activities of daily living (ADL), as well as items related to musculoskeletal pain, mental health, and social functioning. Similar to other musculoskeletal degenerative disorders, such as osteoporosis, osteoarthritis, and spinal canal stenosis, musculoskeletal pain and its psychosocial factors are significant to the impairment of health-related quality of life (HRQOL). The fear-avoidance model proposes a psychological vicious cycle that explains how people develop chronic musculoskeletal pain as a result of the avoidance behavior due to fear of movement-related pain. It has gained empirical application in explaining the development of locomotor disability and subsequent HRQOL as a result of musculoskeletal disorders and the associated chronic pain ^(4)^. People with LS usually experience not only musculoskeletal pain but also instability of their musculoskeletal system, which is related to falling. Falling or the likelihood of falling can lead to negative emotions, such as anxiety and fear, against musculoskeletal pain and instability; this subsequently promotes the vicious cycle involving musculoskeletal pain, instability, and negative emotions, which can make them chronic and severe. In this regard, falling certainly contributes to a reduction in self-efficacy and impairs HRQOL ^(5)^.

In this study, we investigated the relationships between LS, musculoskeletal pain, and other pain-related psychological factors in patients with chronic pain. In congruence with a vicious cycle similar to that of the fear-avoidance model, we conducted a multifaceted analysis on the relevance of the pain, its psychological factors, and LS on HRQOL impairments.

## Materials and Methods

### Participants

Study participants included patients with chronic pain, who initially visited an outpatient clinic in the research consortium (seven Japanese multidisciplinary pain centers) between September 2013 and December 2014. Before their medical examination, they were asked to complete questionnaires listed in the following sections as an initial assessment of their self-reported pain and its-related disability and psychological distress. The local ethics committees approved the protocol of this study [Approval code: 3678-(1)].

### Data collection

The 25-question geriatric locomotive function scale (GLFS-25) is a comprehensive measurement tool of the musculoskeletal system that can screen for LS; its score represents the severity of LS [3]. Each question has an answer based on a scale of 0–5; the total points of the GLFS-25 can reflect no impairment (0 points) to the most severe impairment (100 points). The cutoff point that determines the presence of LS is 16 points. The five subscales of the GLFS-25 are body pain, movement-related difficulty, usual care, social participation, and anxiety. There are questions related to the frequency of exercise with answers that are classified into four categories (0 = do not exercise, 1 = one to three times a month, 2 = one to three times a week, 3 = almost every day). Pain intensity was rated by an 11-point numerical rating scale (NRS: 0 = no pain; 10 = pain as bad as it could be). Psychological distress was measured using the hospital anxiety and depression scale (HADS), which determines the level of anxiety and depression specifically in people with physical health problems ^(6)^. The pain catastrophizing scale (PCS) is a self-reported measurement scale that evaluates catastrophic thinking toward pain ^(7)^. It has three subscales, namely, the magnification, rumination, and helplessness of pain. Furthermore, the pain self-efficacy questionnaire (PSEQ) is used to assess the level of confidence that people with ongoing pain have in performing ADL while in pain ^(8)^. High PSEQ scores are strongly associated with clinically significant functional impairment and provide a useful gage for estimating the prognosis of patients with chronic pain. As a measurement of HRQOL, EQ-5D was used in this study ^(9)^. EQ-5D is a standardized instrument that is applicable in a wide range of health conditions and treatments. EQ-5D can provide a simple descriptive profile and a single index value for health status.

### Procedure

We classified the patients by age (39 years old or younger, 40s, 50s, 60s, and 70 years old or older) and sex. Afterward, we examined the prevalence of LS, trend of the total scores of the GLFS-25, and subscales (body pain, movement-related difficulty, usual care, social participation, and anxiety). Other pain-related rating scales (NRS, HADS, PCS, and PSEQ) and HRQOL (EQ-5D) were also examined.

Based on both the fear-avoidance model of musculoskeletal disorders ^(4)^ and our empirical clinical practice, we created a hypothetical model. In this hypothetical model, we built in LS, musculoskeletal pain, and its psychological factors (measures of self-efficacy, depression, and anxiety about falling in the GLFS-25) and then analyzed the relationships among the factors using a multivariate path analysis. The path analysis is a statistical analysis technique that assumes several causal relationships among variables and makes a causal inference based on a covariance or correlation matrix. It is possible to arbitrarily create a model assuming a freely causal relationship among variables.

### Statistical analyses

Parametric data were compared using Student’s t-test. The prevalence in each group was compared using the Chi-square and Fisher’s exact probability tests and a residual analysis. Nonparametric data, which consist of each evaluation scale, were compared using the Mann–Whitney U test and the Kruskal-Wallis test and Scheffe post-hoc test if significant.

The structural equation model (SEM) for the multivariate path analysis was used to analyze the relevance among LS, musculoskeletal pain, and its psychological factors. The goodness of fit of the SEM models was determined by the Chi-square test, goodness of fit index (GFI), adjusted goodness of fit index (AGFI), comparative fit index (CFI), and the root mean square error of approximation (RMSEA). Acceptable values for the ratio of Chi-square values to degrees of freedom values (CMIN/DF) are less than 2. In addition, the criteria for a good fit between the study data and the model were a GFI, AGFI, and CFI of a least 0.9. In the RMSEA criteria, values up to 0.08 are acceptable, and values equal to or less than 0.05 indicate a good fit. Probability values of 0.05 were considered statistically significant.

SPSS version 22.0 (IBM Co., Armonk, NY, USA) and AMOS version 22.0 (IBM Co., Armonk, NY, USA) were used for statistical analyses.

## Results

We analyzed data from 415 patients (99.0%; total = 419 patients) who answered all the questionnaires. Among the 415 patients with chronic pain, 170 were male, and 245 were female. The average ages were significantly different between the male and female patients (men: 56.2 ± 16.7, women: 51.8 ± 18.9; p = 0.015).

The numbers of the participants classified by age and sex, the prevalence of LS assessed by the GLFS-25, the scores of the subscales of the GLFS-25 (body pain, movement-related difficulty, usual care, social participation, and anxiety), and the trend of the subscales of the GLFS-25, other pain-related rating scales (NRS, HADS, PCS, and PSEQ), and HRQOL (EQ-5D) are presented in Table 1.

**Table 1.** Age, Prevalence of LS, and Trends of the Subscales of the GLFS-25 (Body Pain, Movement-related Difficulty, Usual Care, Social Participation, and Anxiety), Other Pain-related Rating Scales (NRS, HADS, PCS, and PSEQ), and HRQOL (EQ-5D).

		All (n = 415)	Male (n = 170)	Female (n = 245)	p-value
Age		53.6 ± 18.1	56.2 ± 16.7	51.8 ± 18.9	0.015^a^
	≤39	91	29	62	0.001^b^
	40s	81	33	48	0.096^b^
	50s	62	27	35	0.310^b^
	60s	89	38	51	0.168^b^
	≥70	92	43	49	0.532^b^
GLFS-25(+)		337 (81.2%)	136 (80.0%)	201 (82.0%)	0.601^b^
	≤39	69 (75.8%)	24 (82.8%)	45 (72.6%) *	0.291^b^
	40s	64 (79.0%)	26 (78.8%)	38 (79.1%)	0.967^b^
	50s	48 (77.4%)	19 (70.4%)	29 (82.9%)	0.244^b^
	60s	73 (82.0%)	31 (86.1%)	42 (82.3%)	0.925^b^
	≥70	83 (90.2%)	36 (83.7%)	47 (95.9%) **	0.077^c^
Total score of the GLFS-25		33 (19–49)	32.5 (18–45)	34 (20–52)	0.525^d^
Body pain		8 (6–11)	8 (5–11)	8 (6–11)	0.774^d^
Movement-related difficulty		3 (1–4)	3 (1–4)	2 (0–5)	0.621^d^
Usual care		2 (0–6)	3 (0–6)	2 (0–6)	0.381^d^
Social participation		8 (4–12)	8 (4–11)	8 (4–12)	0.331^d^
Anxiety		2 (0–4)	1.5 (0–4)	2 (0–4)	0.484^d^
Average NRS		6 (4–7)	6 (4–7)	6 (5–7)	0.958^d^
HADS		16 (10–22)	16 (11–21)	16 (10–22)	0.966^d^
PCS		35 (27–41)	36 (27–42)	35 (27–41)	0.665^d^
PSEQ		26 (17–36)	26 (17–36)	27 (17–37)	0.552^d^
EQ-5D		0.59 (0.47–0.66)	0.58 (0.47–0.66)	0.59 (0.47–0.66)	0.706^d^

Data were presented as mean ± standard deviation, numbers of patients, number (%), or median (25 and 75 percentiles), as adequate. * p < 0.05 lower than other populations in female patients (residual analysis) **p < 0.01 higher than other populations in female patients (residual analysis) a Student’s t-test b Chi-square test c Fisher’s exact probability test d Mann–Whitney test Abbreviations: LS, locomotive syndrome; GLFS-25, 25-question geriatric locomotive function scale; NRS, numerical rating scale; HADS, hospital anxiety and depression scale; PCS, pain catastrophizing scale; PSEQ, pain self-efficacy questionnaire; HRQOL, health-related quality of life; EQ-5D, EuroQol 5-Dimension survey

Three-hundred thirty-seven patients (81.2%) were determined to have LS (80.0% men and 82.0% women). The prevalence of LS in patients aged 39 years old or younger, 40s, 50s, 60s, and 70 years old or older were 75.8 % (male patients 82.8, female patients 72.6), 79.0 % (male patients 78.8, female patients 79.1), 77.4 % (male patients 70.4, female patients 82.9), 82.0 % (male patients 86.1, female patients 82.3), and 90.2 % (male patients 83.7, female patients 95.9), respectively. There was no significant difference in LS prevalence between male and female patients in each generation. The prevalence of LS among each generation was comparable in the male patients. In the female patients, the prevalence of LS in those 39 years old or younger was significantly lower than that of the other generations (p < 0.05). The highest prevalence was in those 70 years old or older (p < 0.01).

There were no differences in the five subscales of the GLFS-25, other pain-related rating scales (NRS, HADS, PCS, and PSEQ), and HRQOL (EQ-5D) between male and female patients. In the comparisons of each parameter among respective age groups of female, the score of anxiety subscale of the GLFS-25 of the female patients in the 39 years old or younger generation was significantly lower than those of the female patients in their 50s (p = 0.045), 60s (p < 0.0001), and 70 years old or older (p < 0.0001). Among the male patients, there was no significant difference among the generations. In the female patients, the 40s generation demonstrated significantly lower anxiety scores than those of women who were 70 years old or older (p = 0.042). When each parameter was compared between male and female patients by generations, the score of the usual care subscale of the GLFS-25 was higher in men 39 years old or younger (p = 0.015). The score of the anxiety subscale of the GLFS-25 was higher in women in of the 50s generation (p = 0.030).

**Figure 1.**
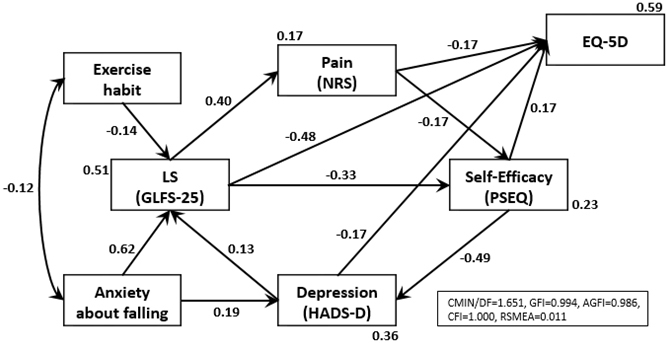
The final model of the relevance of a vicious cycle involving LS, musculoskeletal pain, and its psychological factors. Values on the single-headed arrows are partial standardized regression weights. Values on the double-headed arrows are correlation coefficients. A higher value indicates a stronger causal relationship. Numbers below the rectangles indicate a squared multiple correlation coefficient by the direct-connected upstream variables. For example, 23% of the self-efficacy is explained by pain and the locomotive syndrome (LS). CMIN is a Chi-square statistics comparing the test model and the independent model to the saturated model. CMIN/DF, the relative Chi-square, is an index of how much the fit of data to model has been reduced by dropping one or more paths. Additionally, the goodness of fit of the models was evaluated using the goodness of fit index (GFI), adjusted goodness of fit index (AGFI), comparative fit index (CFI), and root mean square error of approximation (RMSEA).

We created a cause-and-effect relationship model of the parameters and included the total score of the GLFS-25, pain intensity measured by the NRS, PSEQ, PCS, the depression subscale of HADS, anxiety about falling (Question 24 of the GLFS-25), exercise habits (having a couple of opportunities of exercise in a week or more), and EQ-5D. We tested the path analysis by using the maximum-likelihood method of parameter estimation. We examined ten models in reference to previous reports and our clinical experience. The final SEM was shown in Figure 1. The model provided a good fit (χ^2^ [n = 415] = 8.410, CMIN/DF = 1.051, GFI = 0.994, AGFI = 0.980, CFI = 1.000, RMSEA = 0.011). This model demonstrated a vicious cycle among these parameters; each of these was directly associated with an impairment of the HRQOL as assessed by the EQ-5D. Exercise habits had a favorable effect on LS and anxiety about falling in the GLFS-25. Anxiety about falling was unfavorably associated with both the depression scale and the total score of the GLFS-25. In particular, the model using PSEQ demonstrated a higher goodness of fit than that of the PCS (χ^2^ [n = 415] = 9.388, CMIN/DF = 1.173, GFI = 0.994, AGFI = 0.978, CFI = 0.999, RMSEA = 0.020).

## Discussion

The prevalence of LS in the general population is estimated at about 10% ^(10)^. The present study demonstrated that the prevalence of LS in patients with chronic pain was eight times higher than that of the general population. Etiologies of their chronic pain were not confirmed at the time point of survey, and the participants would have varied etiologies of chronic pain (e.g., orthopedic diseases, neurological diseases, and psychological distress). Nonetheless, our present findings clearly confirmed the close relationship between LS and musculoskeletal pain. Since patients with LS often require nursing care services ^(2)^, our data indicate that the risk of patients with chronic pain requiring nursing care services is predictively very high.

Epidemiological studies reveal that generally in Japan and globally ^(11), (12)^, women have more frequent complaints of musculoskeletal pain than men. With regard to musculoskeletal disorders and LS, the differences between the sexes are consistent with those in the general population; the prevalence of LS in women was higher than that in men at all age groups ^(10)^. Unlike the epidemiological population-based studies, our current study only focused on patients with chronic pain and did not reveal a sex-based difference. In contrast, the prevalence of LS increased as the patients aged; this is consistent with the results of a previous epidemiological study in Japan ^(10)^. Several studies suggest that the relationship between psychological distresses, including anxiety, and pain is stronger in women than it is in men ^(13), (14)^. Patients with chronic pain that are accompanied by psychological factors, as it is in fibromyalgia, are often women who are in their late middle ages, and they commonly express anxiety ^(15)^. However, our findings showed that general depressive and anxious states were comparable between male and female patients, although the severity of anxiety increased as the female patients’ age increased.

The usual care score, one of the subscales of the 25-question geriatric locomotive function scale (GLFS-25), was highest in male patients who were 39 years old or younger (p=0.015). Generally speaking, men have a higher pain tolerance than women ^(16), (17)^. However, compared to the male patients, despite an approximately equal level of pain intensity, female patients adapted to pain better, and their pain did not affect their usual care. We could not determine why this sex-based difference is not observed in patients with chronic pain; thus future investigations should be conducted for clarification.

We hypothesized that musculoskeletal pain and LS would form a pattern similar to the one described as the vicious cycle from the well-known fear-avoidance model ^(4)^, rather than a one-way model (i.e., chronic pain might impair LS or vice versa). Take the case of our “vicious cycle” hypothesis of musculoskeletal pain and LS, subsequent to LS emerging and deteriorating, musculoskeletal pain would develop, and movement-related pain would deteriorate. Such musculoskeletal pain can result in general physical inactivity. Next, physical inactivity can cause disuse of the musculoskeletal system and further worsen LS. Ultimately, LS can cause further musculoskeletal pain. Thus, we considered LS and musculoskeletal pain can be linked in a vicious cycle. Furthermore, the original fear-avoidance model ^(4)^ includes musculoskeletal problems as well as some psychological factors, such as catastrophic thinking, fear of movement, and depression. These are clearly deteriorating factors related to pain ^(4), (18), (19), (20), (21)^. Based on our experience, we included pain and psychological factors into the vicious cycle model of LS in our present study. Our results clearly suggest that LS, musculoskeletal pain, and psychological factors are involved in the vicious cycle and the respective building components of each factor independently impaired the HRQOL. Among these, LS directly affected EQ-5D the most. The fear of falling had a considerable impact on LS. Self-efficacy, one of the psychologically modulating factors of pain, had more of an impact on the cycle than pain catastrophizing did when the fitness of two models was compared using either the PSEQ (i.e., self-efficacy) or PCS (i.e., pain catastrophizing). Self-efficacy is the psychological factor that influences exercise habits at every age ^(22)^. Data suggest that adaptive thoughts, including self-efficacy, have greater influence on HRQOL and the degree of ADL dysfunction than maladaptive thoughts and emotions for pain such as catastrophic thinking have ^(23), (24)^. In addition, depression clearly deteriorates ADL and HRQOL. The fear-avoidance model has been studied for its clinical application ^(25), (26)^. Considering these results related to psychological distress, interventions for psychological distress are extremely important when treating LS. Moreover, exercise habits had a favorable effect on the vicious circle, either directly or indirectly, through the relief of anxiety related to falling. Exercise is generally effective against LS. However, since our findings clearly demonstrated that LS and the pain form a vicious cycle, it would be difficult for patients with chronic pain to exercise moderately without pain alleviation. Therefore, treating the pain first would be important.

In conclusion, LS and its associated pain formed a vicious cycle and deteriorated the HRQOL. Therefore, interventions for the musculoskeletal disorder as well as the associated pain and psychological issues and exercise are necessary for the improvement of LS.

Our present study has limitations, which should be considered to resolve in future studies. One is we conducted the questionnaire survey, and our conclusion is based on participants’ subjective complaints. Objective assessments of the musculoskeletal disorder by experienced physicians might more clearly elucidate the vicious cycle and the treatment target. And the other is we did not include patients with LS and/or the musculoskeletal dysfunction, who *did not* complain of chronic pain. We did not compare those with and without pain, and such comparison might lead to novel findings of chronic pain and the LS.

## Article Information

### Conflicts of Interest

None

### Acknowledgement

The authors would like to express their gratitude to Dr. Atsushi Seichi for his permission to use the GLFS-25.

### Approval by Institutional Review Board (IRB)

Approval Code: 3678-(1)
